# 

**Published:** 1890-06

**Authors:** 


### CONTENTS.

COMMUNICATIONS.

Evolution in Dentistry ... 265
Diseases of the Antrum ... 269

SELECTIONS.

A Study of the Relationship between the Eruption of the Permanent Teeth and the Ailments of Late Childhood ... 275
Dental Laws of North Dakota ... 295
About Platinum ... 301
Dangers of Hypnotism ... 303
Chronic Syphilitic Salivation ... 304
Plastic Surgery ... 304
Chicago Dental Society ... 305
Another Warning to Landlords ... 306

PROCEEDINGS.

Annual Meeting of the Northern Ohio Dental Association ... 308
International Congress ... 309

EDITORIAL.

American Medical Association ... 311
Americ n Dental Association ... 312
Association of Dental College Faculties ... 313
National Association of Dental Examiners ... 313
Georgia State Dental Society ... 314
Obituary ... 315

